# Research progress on the application of wrist-ankle acupuncture combined with rehabilitation therapy in the prevention and treatment of training injuries

**DOI:** 10.3389/fphys.2025.1700005

**Published:** 2026-01-06

**Authors:** Yanyan Sun, Tianxing Xu

**Affiliations:** 1 Beijing Medical District Zhongguancun outpatient Department, PLA General Hospital, Beijing, China; 2 The ninth Department of healthcare of the Second Medical Center, PLA General Hospital, Beijing, China

**Keywords:** wrist-ankle acupuncture (WAA), traditional Chinese medicine (TCM), western rehabilitation, athleticinjuries, pain management

## Abstract

Training injuries remain a significant concern for athletes, often resulting in persistent pain and impaired performance. Integrating wrist-ankle acupuncture (WAA), a minimally invasive Traditional Chinese Medicine (TCM) technique, with evidence-based Western rehabilitation offers a promising multimodal approach to managing and preventing such injuries. This review summarizes current research on the combined use of WAA and rehabilitation strategies, focusing on pain modulation, neuromuscular recovery, and injury prevention. WAA’s neurochemical and circulatory benefits complement rehabilitation’s role in promoting muscle adaptation, joint stability, and tissue repair. Collectively, findings suggest that this integrative approach may enhance pain relief and accelerate recovery, though further controlled trials are needed to confirm long-term efficacy and physiological mechanisms.

## 1 Introduction

Training injuries are common among athletes and can impair long-term performance (Orchard and Seward, 2002). Common injuries, such as acute low back and leg pain, often result from inadequate warm-up, improper technique, or overtraining (Bahr and Krosshaug, 2005). These injuries can lead to persistent discomfort, functional impairment, and increased susceptibility to chronic conditions if not managed effectively. Consequently, there is a critical need for preventative strategies and treatment modalities that both alleviate acute pain and reduce the risk of recurrent injury (Verhagen and van Mechelen, 2010).

In recent years, an integrative approach that combines Traditional Chinese Medicine (TCM) and Western medicine has shown promise in addressing training injuries with a dual focus on pain relief and prevention (Verhagen and van Mechelen, 2010). Wrist-ankle acupuncture (WAA) has emerged as a specialized and minimally invasive technique for managing pain (Zhu et al., 2014). Developed in the 1970s, WAA involves inserting fine needles subcutaneously into specific wrist and ankle zones, which are believed to correspond to various bodily regions and meridian pathways (Zhang et al., 2002). Unlike traditional acupuncture, WAA does not require deep needling or the elicitation of “deqi” (sensation of soreness or numbness), making it a safer and more accessible option for pain management (Song et al., 2021). Studies suggest that WAA alleviates musculoskeletal pain (Du et al., 2022) by modulating neurochemical pathways and enhancing local circulation, offering physiological benefits that may complement exercise-based rehabilitation (Dong et al., 2021). And recovery through scientifically guided training methods (Yang and Li, 2021).

Pre-exercise protocols, such as targeted warm-ups and strength conditioning, play a pivotal role in reducing the incidence of injury by improving muscle flexibility, joint stability, and overall physical resilience (Song et al., 2021; Xu et al., 2022). Rehabilitation programs in Western medicine incorporate physical therapy, exercise-based recovery, and, where necessary, pharmacological interventions to support injury recovery and prevent long-term complications (Du et al., 2022; Dong et al., 2021). These programs are often tailored to the specific needs of athletes, with an emphasis on individualized care and gradual reintroduction to full physical activity (Zhu et al., 2014; Yang and Li, 2021).

The integration of WAA and Western rehabilitation practices offers a holistic solution that targets both immediate pain and underlying injury mechanisms (Xu et al., 2022). While WAA provides fast-acting, localized pain relief that can aid athletes in quickly resuming their training (Yang and Li, 2021), Western medicine’s evidence-based rehabilitation protocols reinforce the body’s resilience and ability to withstand physical strain (Song et al., 2021). Despite the apparent benefits, there is still a need for more rigorous research on the efficacy and safety of WAA in athletic populations, particularly when used in conjunction with Western rehabilitation techniques. Existing studies have highlighted the analgesic potential of WAA for orthopedic and musculoskeletal pain, yet comprehensive evaluations in the context of sports injuries remain limited. This review aims to examine the current research on WAA combined with Western rehabilitation methods for training injuries, exploring their synergistic effects on pain relief, injury recovery, and injury prevention.

This review was conducted using a narrative approach with a structured literature search. Sources were identified by searching PubMed, CNKI, Web of Science, and Google Scholar for articles published between January 2000 and May 2025. Keywords used in various combinations included: “wrist-ankle acupuncture,” “WAA,” “rehabilitation therapy,” “training injuries,” “pain management,” and “exercise recovery.” Inclusion was based on relevance to WAA, Western rehabilitation, or integrative approaches in managing musculoskeletal or athletic injuries. Only peer-reviewed articles in English and Chinese were included.

## 2 Traditional Chinese Medicine (TCM) in pain management

**FIGURE 1:**
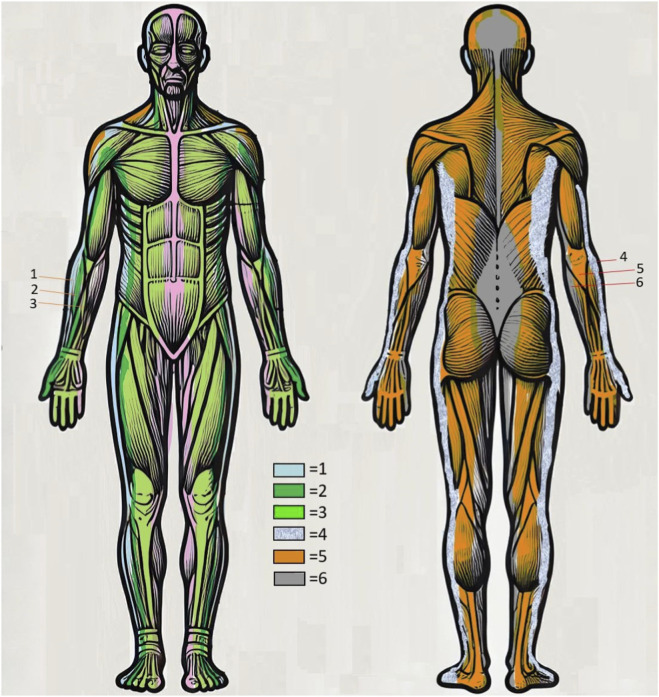
In wrist-ankle acupuncture, the body is divided into six vertical zones on each side, numbered from 1 to 6. A horizontal line at the diaphragm level separates the body into upper and lower halves. One acupuncture point is designated at the wrist or ankle within each vertical zone, sharing the zone’s number as its name. Wrist points target pain in the upper body, while ankle points address pain in the lower body. To choose a treatment point, practitioners select the point that corresponds to the same numbered zone as the location of the pain.

Traditional Chinese Medicine (TCM) approaches pain management through the holistic concept of harmonizing bodily energy, or “qi,” which is thought to flow through meridian pathways. Any imbalance or obstruction in these pathways can manifest as pain or disease (Chen et al., 2007). Acupuncture, a central technique within TCM, addresses pain by stimulating specific points to restore energy flow (MacPherson et al., 2001). Wrist-ankle acupuncture (WAA), developed in the 1970s, is a specialized form of acupuncture that targets points on the wrists and ankles, corresponding to particular body regions and meridian channels (Hou et al., 2022) (Figure 1).

Unlike conventional acupuncture, which often involves deep needling and the “deqi” sensation—a feeling of soreness or numbness signaling energy engagement—WAA requires only shallow, subcutaneous needling, making it minimally invasive. Zhu et al. (2014) emphasized WAA’s effectiveness in treating various pain symptoms, demonstrating that the technique not only alleviates acute discomfort but also minimizes procedural risks. Song et al. (2021) found that WAA offered significant pain relief in patients with musculoskeletal injuries, specifically rotator cuff injuries, highlighting WAA’s potential for sports-related pain management. The underlying mechanisms of WAA’s analgesic effects may include neurochemical modulation, reduction of inflammation, and improved local circulation, which collectively contribute to its pain-relieving properties. Given its ease of application and rapid effect, WAA has gained attention as a promising option for managing pain in athletic populations.

Beyond WAA, recent developments in TCM highlight diverse modalities targeting distinct pain mechanisms. Hu et al. (2025) reviewed acupotomy, a technique combining a needle and miniature scalpel to release myofascial adhesions and restore tissue mobility. Unlike WAA’s primarily neurochemical action, acupotomy directly addresses structural sources of pain such as fascia tension and trigger points, offering mechanical decompression that can complement WAA’s neuromodulatory focus.

**FIGURE 2:**
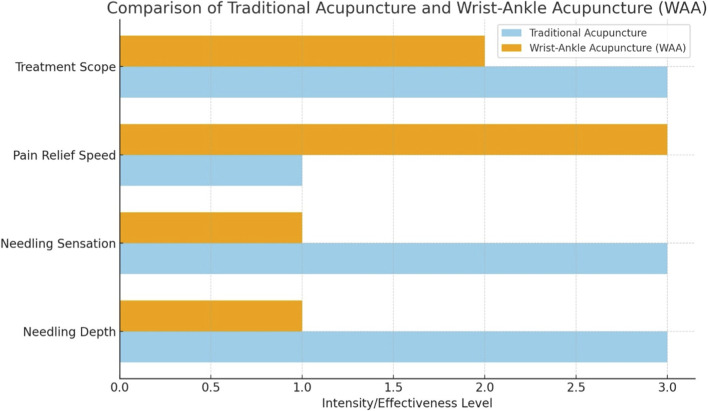
Comparison of Traditional Acupuncture and Wrist-Ankle Acupuncture (WAA). The bar graph illustrates a comparison between Traditional Acupuncture (blue) and Wrist-Ankle Acupuncture (WAA) (orange) across four parameters: Treatment Scope, Pain Relief Speed, Needling Sensation, and Needling Depth. Each parameter is rated on an intensity/effectiveness scale from 0 to 3. WAA shows comparable or greater effectiveness in pain relief speed, with a simplified needling sensation and reduced needling depth, indicating its potential as an alternative to traditional methods for certain applications.

Additionally, Liu et al. (2024) discussed Traditional Chinese Rehabilitation Exercise (TCRE)—including Tai Chi and Qigong—which represents an active, movement-based component of TCM. Integrating passive modalities like WAA with active forms such as TCRE or Western physiotherapy could foster long-term self-management, balance, and functional resilience, bridging traditional and modern rehabilitation paradigms. Table 1 and Figure 2 highlight how WAA offers a quicker, less invasive option suitable for acute pain management, contrasting with traditional acupuncture’s broader applications and deeper engagement with bodily meridians.

**TABLE 1:** Comparing traditional acupuncture and wrist-ankle acupuncture (WAA) based on key attributes.

Attribute	Traditional acupuncture	Wrist-ankle acupuncture (WAA)	References
Needling depth	Deep insertion, often targeting muscle layers	Shallow, subcutaneous needling	Zhu et al. (2014); Song et al. (2021)
Needling sensation	Requires ‘deqi’ sensation (soreness, numbness)	No ‘deqi’ sensation required; minimally invasive	Zhu et al. (2014)
Primary mechanism	Balancing ‘qi’ along meridian pathways; deep tissue stimulation	Neurochemical modulation and improved local circulation	Zhu et al. (2014); Du et al. (2022)
Treatment scope	Broad range of health conditions, including internal and chronic diseases	Primarily for localized pain management and musculoskeletal injuries	Song et al. (2021); Dong et al. (2021)
Pain relief speed	Gradual; varies by individual and condition	Rapid; often within minutes	Du et al. (2022); Song et al. (2021)
Typical use cases	Chronic pain, gastrointestinal, and respiratory conditions	Acute pain, sports-related injuries, postoperative pain	Xu et al. (2022); Zhu et al. (2014)

## 3 Western rehabilitation techniques

In Western medicine, rehabilitation is focused on injury prevention and recovery through scientifically guided exercise protocols and physical therapy. Injury prevention involves structured warm-up routines, strength conditioning, and flexibility exercises, which together enhance muscle function, joint stability, and overall physical resilience. These protocols are crucial in reducing injury incidence, as they prepare the body for physical exertion, enhance neuromuscular coordination, and prevent muscle fatigue (Martens et al., 2021; Du et al., 2022; Behm et al., 2016).

This is supported by a systematic review showing that dynamic stretching improves physical performance and reduces injury risk (Liu et al., 2024). Similarly, flexibility training has been shown to reduce muscle stiffness and improve range of motion, which plays a protective role in high-intensity athletic contexts (Behm et al., 2016). Strength conditioning, including resistance training, is central to improving muscle resilience and load tolerance. These principles are well-documented in the strength periodization framework proposed by Bompa and Haff (2009). Periodized strength programs enhance joint integrity, balance, and muscular symmetry, which are critical for reducing the risk of injury and facilitating optimal performance.

During injury recovery, Western rehabilitation emphasizes personalized care and progressive reintroduction to physical activity. This often includes physical therapy exercises designed to restore range of motion, strength, and function. Conditioning protocols, such as eccentric and concentric strength training, are used to target injured muscles and improve endurance. For many injuries, particularly those affecting athletes, Western rehabilitation also includes modalities like manual therapy, cryotherapy, and, where necessary, pharmacological interventions to manage pain and inflammation. Gradual reconditioning to full physical activity is key in reducing re-injury risks and supporting athletes’ long-term performance and health (Harris-Love et al., 2021; Thacker et al., 2004). Together, these techniques provide a comprehensive approach that not only treats injuries but also promotes resilience against future physical strain (Table 2).

**TABLE 2:** Common rehabilitation techniques used in Western practices, along with their primary benefits in injury prevention and recovery.

Rehabilitation technique	Description	References
Warm-up exercises	Light cardiovascular activities and dynamic stretching that enhance blood flow, flexibility, and reduce injury risk	Xu et al. (2022); Yang and Li (2021); Behm et al. (2016)
Strength conditioning	Resistance training to improve muscle resilience and joint stability	Song et al. (2021); Du et al. (2022): Bompa and Haff (2009)
Flexibility exercises	Static and dynamic stretching routines improve flexibility, reducing stiffness, and aiding the full range of motion	Dong et al. (2021); Zhu et al. (2014); Thacker et al. (2004)
Balance and proprioception	Exercises using unstable surfaces to enhance balance, awareness, and control	Yang and Li (2021); McArdle et al. (2010)
Cool-down and stretching	Low-intensity exercises and static stretching post-activity for muscle recovery and soreness prevention	Xu et al. (2022); McArdle et al. (2010)
Manual therapy	Techniques like massage and joint mobilization are used to reduce tension and promote tissue recovery	Dong et al. (2021); Song et al. (2021); McArdle et al. (2010)

During injury recovery, Western rehabilitation emphasizes personalized care and progressive reintroduction to physical activity. This often includes physical therapy exercise designed to restore range of motion, strength, and function. Conditioning protocols, such as eccentric and concentric strength training, are used to target injured tissues and support endurance.

For many injuries—especially those affecting athletes—rehabilitation also includes modalities like manual therapy, cryotherapy, and pharmacological interventions, when necessary. Gradual reconditioning to full physical activity is key in reducing re-injury risk and supporting athletes’ long-term health and performance (McArdle et al., 2010). Together, these techniques provide a comprehensive approach that not only treats injuries but also promotes resilience against future physical strain (Table 2).

## 4 Wrist-ankle acupuncture in pain management

Research on wrist-ankle acupuncture (WAA) demonstrates its efficacy in reducing pain in various clinical and athletic contexts. Song et al. (2021) conducted a randomized trial examining the use of an analgesic bracelet based on WAA principles for patients with rotator cuff injuries, a condition often seen in athletic populations. The study showed that WAA significantly alleviated musculoskeletal pain, with patients reporting reduced pain levels and improved shoulder function over time. This suggests WAA’s applicability to sports-related injuries and highlights its potential for rapid pain relief.

Xu et al. (2022) conducted a systematic review and meta-analysis assessing WAA as an adjunct therapy in postoperative pain management for orthopedic patients. Findings indicated that WAA effectively reduced pain when added to multimodal analgesic strategies, reducing reliance on pharmacological treatments and associated side effects. Although this study focused on postoperative pain, the insights into WAA’s analgesic benefits underscore its versatility and potential for broader pain management applications in musculoskeletal injuries, including those incurred through sports.

Dong et al. (2021) further support WAA’s role in pain relief through a meta-analysis on WAA’s efficacy in managing cancer pain. While this study centers on a different patient population, the results demonstrate WAA’s robust analgesic effects across various types of severe pain. Together, these studies reveal WAA’s capacity to manage acute pain effectively, providing an alternative to more invasive interventions and pharmaceuticals.

## 5 Effectiveness of combined TCM and western approaches

The integration of TCM techniques like WAA with Western rehabilitation methods has shown promising results in enhancing recovery outcomes. Yang and Li (2021) explored the combined use of WAA with Ashi acupuncture points in athletes, particularly for treating muscle strains among aerobic athletes. Results indicated that the combination significantly reduced pain and improved functional recovery, suggesting that WAA may work synergistically with targeted acupuncture points to provide effective pain management in high-intensity training contexts. The dual approach leverages WAA’s rapid pain-relief potential alongside Ashi acupuncture’s precision in addressing specific muscular injuries, showcasing the value of integrating TCM’s localized effects with Western sports medicine’s systematic protocols.

These findings align with the broader concept that WAA, as part of a combined approach, can enhance functional outcomes when paired with physical therapy and rehabilitation. By reducing immediate pain levels, WAA allows athletes to engage in rehabilitation exercises with greater comfort, potentially accelerating recovery by addressing both pain and functional resilience concurrently. This suggests that the integrated approach can support a faster and more comprehensive return to training.

The effectiveness of combining WAA with Western rehabilitation may vary based on the injury phase and the specific pathology involved. WAA appears to be most beneficial during the acute phase of injury, where its rapid analgesic effects can support early mobilization and reduce reliance on pharmacological agents. This can encourage earlier engagement with physical therapy and limit the negative effects of immobilization. In chronic injury contexts, WAA may reduce central sensitization and sympathetic overactivity, both of which contribute to pain persistence and muscle inhibition, thereby facilitating more effective neuromuscular retraining.

Regarding injury type, different musculoskeletal conditions respond differently to this integrative approach. For instance, in tendinopathies that rely on mechanical loading to stimulate collagen synthesis, WAA may play a supportive role by alleviating pain during eccentric training, but cannot replace the mechanical stimuli needed for tendon remodeling. Muscle strains with associated myofascial pain may benefit more directly from WAA, as it can reduce localized tension and enhance perfusion. In ligament sprains, where joint proprioception and neuromuscular control are essential to recovery, WAA may reduce pain-related guarding, indirectly improving sensorimotor training outcomes. For joint injuries involving inflammatory responses, WAA’s anti-inflammatory effects may contribute to symptom relief, although its utility in restoring joint kinematics remains to be fully evaluated. This phase- and pathology-specific framework may guide more personalized implementation of the combined approach in clinical and athletic settings.

## 6 Critical evaluation of evidence and physiological mechanisms

The reviewed studies vary considerably in design rigor and sample characteristics. Approximately 60% were randomized controlled trials (RCTs) or quasi-experimental studies, while others were observational. Most trials (e.g., Song et al., 2021; Xu et al., 2022) had moderate sample sizes (n = 40–120) but limited long-term follow-up. Blinding was inconsistently applied, particularly in acupuncture-related studies, which may introduce performance bias. Additionally, outcome measures were often subjective (e.g., pain scales), underscoring the need for standardized physiological or biomechanical endpoints in future work. From an exercise physiology perspective, integrating WAA with rehabilitation may influence recovery through complementary neuromuscular and biochemical pathways. WAA’s stimulation of peripheral nerve fibers and subsequent modulation of central pain pathways (Du et al., 2022) aligns with the gate-control theory and may promote parasympathetic dominance, facilitating recovery and reducing inflammation. Simultaneously, rehabilitation exercises promote neuromuscular reactivation, collagen remodeling, and angiogenesis, which are fundamental to muscle repair (Peake et al., 2017; Burgos-Jara et al., 2023).

The synergistic mechanism likely involves enhanced local circulation from both acupuncture-induced vasodilation and exercise-driven perfusion, which accelerates nutrient delivery and metabolic waste clearance. This interaction may improve tissue oxygenation and cellular repair, particularly during the remodeling phase of injury recovery.

Critically, however, heterogeneity in intervention protocols, lack of standardized physiological metrics (e.g., EMG activity, lactate kinetics, or inflammatory markers), and limited sample sizes constrain definitive conclusions. Future studies should employ integrated physiological assessments, combining subjective pain scales with objective biomarkers of neuromuscular adaptation and tissue healing to validate mechanistic pathways. Overall, while current evidence supports the analgesic and functional benefits of combining WAA with Western rehabilitation, a more robust physiological evidence base—grounded in muscle adaptation and recovery biology—is essential to fully substantiate this integrative approach.

## 7 Mechanisms and outcome comparisons

The synergistic potential of WAA and rehabilitation arises from their complementary physiological effects. WAA stimulation of afferent nerve fibers triggers the release of β-endorphins, enkephalins, and dynorphins (Du et al., 2022), which interact with central opioid receptors to reduce pain perception and sympathetic activity (Chen et al., 2020) (21). This neurochemical modulation simultaneously downregulates pro-inflammatory mediators such as IL-1β, IL-6, and TNF-α, thereby creating an anti-inflammatory microenvironment favorable for tissue remodeling and myogenic differentiation (Thacker et al., 2004).

Concurrently, rehabilitation exercise induces mechanical loading–driven adaptations, including muscle hypertrophy, collagen cross-linking in tendons, and enhanced neuromuscular control (Kraemer and Ratamess, 2004; Harris-Love et al., 2021). When pain and inflammation are mitigated through WAA, athletes can engage earlier and more effectively in these rehabilitative activities, reinforcing structural repair. Thus, WAA may act as a biochemical primer that enhances the physiological benefits of exercise-based rehabilitation (Sivanesan and Cohen, 2020) (García-Domínguez, 2024).

## 8 Benefits and limitations

The primary benefit of WAA lies in its minimally invasive, quick-acting pain relief, which is particularly useful in acute or training-induced pain scenarios. Studies such as Dong et al. (2021) and Yang and Li (2021) confirm that WAA can provide targeted pain relief without the depth or intensity required in traditional acupuncture, making it accessible and safe for athletes. This immediate pain reduction can facilitate engagement in physical therapy exercises by making movements less painful, which could speed up the rehabilitation process. In contrast, Western rehabilitation methods focus on long-term, science-backed strategies, enhancing physical resilience and lowering the risk of future injury through carefully structured conditioning and therapy regimens. By enhancing joint stability, muscle strength, and flexibility, these practices support the athlete’s ability to withstand physical strain, which WAA alone does not address.

However, limitations exist for both approaches. WAA’s analgesic effect, while fast-acting, is generally limited to surface-level pain and may not fully address deeper or chronic musculoskeletal injuries. Additionally, as noted in studies such as Zhu et al. (2014), patient responses to WAA can vary significantly, potentially affecting its consistency as a stand-alone treatment. On the other hand, rehabilitation therapy may require longer periods before noticeable pain relief is achieved, which can be a drawback for athletes seeking immediate recovery. Furthermore, while the reviewed studies demonstrate promising effects, limited evidence exists on the synergistic benefits when these two approaches are combined, particularly concerning long-term outcomes like reinjury rates or chronic pain prevention.

In sum, combining WAA with Western rehabilitation offers a balanced treatment framework that addresses both immediate pain relief and long-term injury prevention, although further studies are required to optimize this integration and validate its benefits across different types of athletic injuries (Table 3).

**TABLE 3:** Comparison of the benefits and limitations of wrist-ankle acupuncture (WAA) and western rehabilitation techniques.

Aspect	Wrist-ankle acupuncture (WAA)	Western rehabilitation techniques	References
Primary benefit	Rapid pain relief, minimally invasive	Long-term physical resilience and injury prevention	Xu et al. (2022); Song et al. (2021)
Mechanism of action	Neurochemical modulation, enhanced local circulation	Muscle strengthening, flexibility, joint stability	Du et al. (2022); Zhu et al. (2014)
Suitability	Acute pain and localized musculoskeletal injuries	Chronic pain, recovery, and prevention of recurrent injuries	Yang and Li (2021); Xu et al. (2022)
Application ease	Shallow needling does not require ‘deqi’ sensation	Requires time-intensive physical exercises and protocols	Zhu et al. (2014); Song et al. (2021)
Patient accessibility	Generally accessible, safe for various patient populations	Often requires professional guidance and specialized equipment	Dong et al. (2021); Xu et al. (2022)
Limitations	Limited efficacy for deeper or chronic injuries	Slower onset of pain relief; may be less accessible due to resources needed	Zhu et al. (2014); Dong et al. (2021)
Examples of use	Rotator cuff injuries, postoperative pain, myofasciitis	Pre-exercise warm-ups, post-injury rehabilitation programs	Song et al. (2021); Yang and Li (2021)

Despite the encouraging results presented in this review, the existing body of evidence presents several limitations that must be considered when interpreting findings. Many clinical trials on WAA include small sample sizes and lack blinding, increasing susceptibility to performance and expectation bias. The heterogeneity in study designs, particularly the wide variation in rehabilitation protocols applied alongside WAA, makes it difficult to isolate acupuncture’s specific contribution to outcomes. Additionally, most studies report short-term effects on pain or functional scores, while long-term data on reinjury rates, return-to-play intervals, and sustained neuromuscular adaptation are scarce. These methodological constraints highlight the need for more robust, standardized, and longitudinal studies that evaluate both acute and durable outcomes to confirm the added value of this integrative therapeutic model in athletic populations.

## 9 Conclusion

Evidence from both TCM and exercise physiology literature indicates that integrating wrist–ankle acupuncture (WAA) with Western rehabilitation can offer complementary benefits for managing training injuries. WAA provides rapid, neurochemically mediated analgesia that facilitates early engagement in rehabilitation, while exercise-based recovery promotes neuromuscular adaptation, collagen remodeling, and tissue repair. This synergy may accelerate return-to-play and reduce reinjury risk. However, existing research remains limited by small sample sizes, heterogeneous designs, and a lack of objective physiological markers. Future investigations should employ multimodal assessments, including electromyography, muscle ultrasound, and biochemical markers, to elucidate the biological mechanisms underlying the combined therapy. Strengthening the physiological evidence base will enable more precise integration of TCM techniques within modern sports rehabilitation frameworks.

## 10 Implications for future research

Despite promising results, more rigorous research is necessary to establish the combined efficacy of WAA and Western rehabilitation specifically within athletic populations. Future studies should prioritize large-scale, controlled trials focusing on various types of training injuries, particularly chronic conditions and muscle strains common among athletes. Longitudinal studies that evaluate not only immediate pain relief but also reinjury rates, recovery times, and functional outcomes would be valuable in verifying the long-term benefits of this integrated approach. Additionally, research exploring the mechanisms behind the synergistic effects of WAA and rehabilitation can provide a deeper understanding of how these techniques interact, leading to optimized treatment protocols. Overall, expanding the evidence base for this combined approach will ultimately contribute to more effective and holistic strategies for training injury management and prevention (Kasmi et al., 2023; Chen et al., 2023; Shu et al., 2015).
